# Women’s Expressed Motivational Factors for Participation in Aquarobics Classes

**DOI:** 10.3390/ijerph19095274

**Published:** 2022-04-26

**Authors:** Janet L. Currie

**Affiliations:** School of Education, Macquarie University, Sydney 2109, Australia; janet.currie@mq.edu.au

**Keywords:** older women, leisure, physical activity motivation, aquarobics, women’s health, wellness, subjective wellbeing, social connectedness

## Abstract

Leisure provides a vehicle for women to develop friendships in later life, yet few studies have explored older women’s experiences of social and emotional connections with leisure. This article provides insights into the perceived benefits a group of older women state they gain from participation in aquarobics for leisure. The main themes to emerge from the focus group interview data (*n* = 19 women, $\bar{x}$ age = 75 years), illustrating the key motivational factor for participation in the classes, included an individual desire to improve one’s overall fitness. Participants also expressed a strong sense of belonging from being able to socialize after the activity, and highly valued the welcoming atmosphere and feeling of comradery involved. The aquarobics instructor was noted as performing a very important role in ensuring delivery of an enjoyable class experience where participants experienced variety, had fun, and felt happy taking part. For this group of women, aquarobics forms a health promoting leisure context offering an important space for gaining subjective wellbeing, building social connectedness and resisting the dominant ideology of aging.

## 1. Introduction

Research suggests that while leisure provides a vehicle for women to develop friendships in later life, there is a paucity of research exploring older women’s perceived experiences, including potential for social connections, in leisure settings [1]. The opposite of loneliness, ‘social connectedness’ is a basic human need that may influence health and wellbeing for older adults. It includes a positive subjective evaluation by which an individual believes they are experiencing meaningful, close, and constructive relationships with other individuals, groups, or society [2].

Research points to older adults feeling less supported in availability of leisure programs. Interventions have been recommended to be developed which combat risk of loneliness and promote social connectedness [3,4,5,6]. However, this group has rarely had the opportunity to express their own views about the concept [7]. Greater research is needed to better understand how positive social connections of older people in community leisure activities may be achieved [8]. Further, involving participants at the ‘grass roots’ level in discovering their feedback, motivations, and any barriers regarding early experiences in programs can help ensure that future health promotion design and implementation better meets their expressed needs [9,10,11].

As women’s participation in physical activity significantly declines post-menopause, further options are needed in the community to help improve participation rates [12,13]. Towards this end, future research is needed into reliable predictors of exercise adherence in older people to better understand antecedents and motivational factors, and to help facilitate access to physical activity, particularly for the sedentary [14], frail, or recently bereaved [15]. One such potential avenue for research is that of ‘Aquarobics’. It is surprising that while aquarobics is one of the few leisure activities able to be engaged in comfortably and with high levels of self-efficacy by this group, there is a distinct paucity of what the experience means for participants. With older people not participating in sufficient exercise for health benefits, aquarobics may provide a viable option or avenue to help increase levels of physical activity [16].

Therefore, the purpose of this article is to draw attention to the experiences of older women’s participation in leisure and the reasons they give for taking part. According to Tumosa (2022):

*Promotion of health and wellness interventions for older adults is important in controlling the onset and progression of disabilities and disease. An initial step in determining how to promote health and wellness is to consult with older adults directly*.

[17]

The article fills a gap in the past research in attempting to consult directly with a group of older women to gain their expressed motivations for engagement in aquarobics for leisure.

## 2. Material and Methods

This qualitative study involved a purposeful sample of 19 women ($\bar{x}$ age = 75 years) engaged in focus group interviews, with the goal for each participant to share their own perspective of the individual motivational reasons for taking part in the classes. Focus groups are an appropriate research methodology to employ when attempting to gain insight into attitudes and behaviors:

*The focus group is a method that capitalizes on the discussion generated among participants. The participants are encouraged to not only respond to the moderator but also to the anecdotes conveyed by other participants and to engage in further reflection of personal experiences as others speak. The naturally occurring discourse or spoken words constitutes the data analyzed. These data are considered rich (i.e., descriptive and elaborate) because they go beyond superficial explanations. Instead, the data are representative of genuine attitudes, beliefs, feelings, and the justifications of the perspectives. In a way, the focus group methodology presents researchers with access that other methods cannot*.

[18] (p. 579)

Institutional ethics approval was gained, with all participants completing written informed consent to take part, with all names and identities de-identified. A semi-structured interview approach was used, guided by a schedule containing open-ended questions designed to elicit those factors the women stated motivated them to take part in the classes. The focus group transcripts were transcribed verbatim, and content analyzed line by line using the grounded theory method outlined by Corbin and Strauss [19]. Data saturation was deemed to occur when no new themes emerged from the data [20]. Thus, the explanations of the main findings are based on the women’s own rich descriptions of their individual lived experience [21].

The focus groups were conducted by the researcher in a convenient, naturalistic leisure setting (Sydney, Australia), where participants normally met for coffee to socialize after the aquarobics class. Each typical visit to the leisure setting for a participant involved active participation in an aquarobics class for 50 min, followed by changing into dry clothes in a private changeroom area and moving to a post-class café area, featuring lots of large circular tables, chairs, and a nearby cafeteria selling coffee, tea, and refreshments.

An aquarobics class is sometimes referred to as ‘water aerobics’, adapting various exercises and movements from land-based classes to the pool or water-based environment. Participants are normally able to support themselves standing, balancing with greater confidence, and exercise at a mid-chest water depth level. Aquarobics offer cardiovascular fitness training, plus muscular conditioning due to the resistance of moving against the water’s pressure and gravity. Aquarobics produce less impact on the joints due to the supportive quality of water, and are safer, as the participant is less likely to injure themselves from falling. Aquarobics for older adults may be structured at a lower degree of intensity or offer options to each exercise, as may suit the class’s needs.

The study is underpinned by the principles of health promotion, whereby empowered individuals, facilitated by supportive environments, may engage in behaviors and supports which improve their individual sense of wellbeing and control [22]. Additionally, the positive impact of social connection theory is recognized, whereby individuals high in social connection may change their cognitive interpretation of stress through emotion regulation, decreasing the stress-induced cortisol levels, exerting a calming effect on the nervous system [23]. The senses of social connection serve as a protective factor that provides a significant positive correlation with emotional well-being [24,25]. The next section outlines the main findings to arise from the focus groups.

## 3. Results

The first theme to emerge from the data was illustrated by a feeling of motivation to gain physical fitness benefits. This related to individuals wanting to gain perceived physical and functional improvements to their body as a result of taking part in the classes.

### 3.1. Physically Mobile: I Want to Get Fit and Feel the Difference in My Body

Typical responses included feeling more mobile, greater flexibility, and less stiffness:

*I come to keep fit and active. That’s the main thing. If you don’t come for a week or so you can feel the difference, everything stiffens up. You know, when you come you can feel the difference. And your coordination as well. So, you keep moving. ‘Use it or lose it’, as they say*.

(Del)

*I come because I just want extra strength and to be fitter than I have been. Just so I feel better by that. Because as you get older, you start to feel more aches and pains. I think taking stock of your health…just to be fitter health wise to be able to travel more*.

(Betty)

*I’ve got arthritis in just about every joint, so it’s (aquarobics) what keeps me active. My knees were so swollen about 5 years ago and because I come so regularly, they’ve gone right down*.

(Meg)

A few individuals also expressed an underlying motivation to achieve greater ‘longevity’, typified in this response:

*Well, I come to get fit. ‘Cause I live on my own and I want to keep fit and live a bit longer–worry my children!*

(laughs) (Donna)

### 3.2. Mental Benefits: It Energizes You

This factor was characterized by a feeling of being ‘energized’, happier, or less stressed:

*It definitely energizes you. Any exercise energizes you and you feel the benefit. The more you’re fit, the more you want to be fit. Mentally, I come some days and I’m really stressed about something, or a little bit sad or lonely and I come and do the class and I walk out with a smile*.

(Zara)

*I come along because I want to get more flexible and feel fitter, but overall, in general, just in having more energy is the main reason and I do find I have more energy by doing the aquarobics exercise. You don’t feel as sluggish, you feel more energized to do other things in your life*.

(Nerina)

*I can feel the investment. It gives me the incentive because you’ve got endorphins, it’s a ‘feel good’ feeling and your body loves you back. I’m having fun enjoying myself every class*.

(Evie)

### 3.3. Social Benefits: ‘We’ve Developed Quite a Nice Social Outlet’

Aquarobics was characterized as a being source of comradery and belongingness. All participants reinforced the important feature of accessing social support and agreed that friendship is a key reason motivating them to attend. Meg explained this concept:

*I think it’s a social thing we’ve developed quite a nice social outlet. There’s about 14 or so, there used to be 9 of us. We break up into little groups for coffee (after the class). With our group, if somebody’s got a problem, they air it amongst other things and we’ve just all met over the last 3 or 4 years*.

It is quite an inclusive atmosphere in welcoming newcomers, as Meg also mentioned:

*I’ve seen people that look like they’re new and they look a bit lonely and I usually say, ‘hi’*.

With the conversational discussion style of focus groups, issues, and concerns were also raised related to factors influencing motivational levels and attitudes towards the classes. Thus, a range of additional themes emerged representing conditions or factors perceived as influencing motivational reasons for attending. These are presented in the next section.

### 3.4. The Critical Role of the Aquarobics Instructor

All but two of the participants discussed the important role of the aquarobics instructor as being highly influential in whether the class was enjoyed or not. Donna explained this concept:

*I would say [attending] depended on the instructor. They have to make it enjoyable. If you haven’t got a good instructor for the day, you haven’t worked. If they’re good, they make it enjoyable, they work you hard and you’re not looking up at the clock. Someone who connects with our age.*

According to Evie, “Your body needs freshness every time”. The instructor is responsible for the music selection and delivery of the range of exercises, as described in these responses:

*I keep coming here because each class is unique*.

(Caitlyn)

*What I like about this class is the variety, there’s such a variety. Every week there’s something different*.

(Fiona)

Lori described the instructor as being the class ‘motivator’:

*A motivator. Someone who motivates you. They exude that persona.*

*They care about you.*

However, she stated that if the instructor didn’t offer her variety in the regular class format it would even influence her attendance:

*The only reason I would not come would be if Sally’s on. She just drives me crazy so I’d rather not come. I don’t like the instructors who don’t change their class routine. Sally’s done the same routine for 2 years. I’ve spoken to her when after a month’s holiday, she did exactly the same kind of class. I went to her after the class and I said, “Sally, I am so bored with your class. I don’t want to be unpleasant, but please change”, and she did.*

Evie concurred with this view:

*Some of the classes are getting a bit boring because the instructor repeats the same thing over and over. So, the classes lose that motivational aspect and incentive of freshness*.

(Evie)

Further comments illustrating attitudes towards the pivotal role of the class instructor influencing motivation and enjoyment levels included:

*The attitude of the person [is important], and their choice of music. If they look bored….*

(Del)

*It’s because she’s [the instructor] talking to us. There’s a connection. I think she gives the feeling this teacher understands and cares. Yeah, it’s a good thing. That’s important… I love it, I really enjoy it. I come here to entertain myself and have a good time and have fun. I like happy classes and then you come here and look forward to it. And that’s the main thing. No pressure, not forcing yourself, push, push. I think that’s defeating the purpose. To feel ok in the class and to feel ok if you want to take a little break or something, then join back in. It’s for your wellbeing, so you’ve got to pick something you enjoy and have fun. Also, this class is very unique because we have some variation in each of the classes and today, we even had the chance for some relaxing meditation. And I think because you have a variety and it caters for all levels, you cannot be left behind. I find this is friendly and no intimidation. The instructor gives people the comfort and reassurance they belong, and if they need a rest, for example, they just take it. I think it’s good for beginners who are trying to keep up and think the instructor cares*.

(Evie)

*I think the class has to be enjoyable. If it’s not enjoyable, I’m not going to come. I think if the class has got good music, and the instructor’s got a good attitude, that’s important. I don’t like classes that are just a hard slog, I haven’t got the strength. There has to be an element of fun, and if you haven’t got that I won’t come. The instructor is important. I think not too serious. They’ve got to have a good personality. You want to get that feeling going in, otherwise it’s too much pressure or something*.

(Oprah)

### 3.5. Other Factors

The class setting and its facilities were deemed as other factors helping to influence a positive outcome and motivating participants to attend. Typical responses included:

*We need the club facilities like coffee, cake, tables, seating*.

(Fiona)

*We can do it in any weather here, it’s indoors*.

(Del)

*And you can park underneath so don’t have to worry about the rain*.

(Meg)

Polly explained that exercising in the water helped her feel comfortable, so more relaxed and willing to participate:

*I like how when you come you can work at your own pace, you know you can be the slowest or you can be the quickest, you know, we all fit in. You can be the skinniest, the fattest, non-one knows as we’re in the water, but you feel comfortable here so there’s no pressure. I’m very comfortable here. I feel I belong. I’m not going to stand out because I can’t do something.*

Del mentioned how the supportive nature of exercising in the pool aided her in completing the activity:

*The water supports your body so that you’re not wearing out joints or jarring.*

The key link that could be established between the main themes to arise was that of participants wishing to achieve age-defying wellbeing in a socially supportive environment. For this group of women, aquarobics forms a health promoting leisure context offering an important space for gaining subjective wellbeing, building social connectedness, and resisting the dominant ideology of aging [5]. The next section discusses the findings.

## 4. Discussion

Wishing to enhance one’s own level of physical fitness was expressed by the women as a key motivational factor for attending the class. This reason related to participants wishing to move freely and anticipating ongoing mobility. Participation in exercise is associated with improvement of physical health [26,27,28,29]. Arthritis Australia recommends water exercise as an excellent way for people to build strength, ease stiff joints, and relax sore muscles [30]. Aquarobics or water-based exercise programs have been shown to be effective in enhancing self-efficacy, decreasing pain, and improving depression levels, body weight, and blood lipid levels even in patients with osteoarthritis [31]. The Aquarobics participants’ positive anticipation of these benefits may be described as a form of intrinsic motivation (positive internal feelings associated with exercise) which enable or facilitate ongoing exercise adherence by participants [32]. These beliefs are backed up by strong external evidence stating participation in exercise is a strong predictor of the health for women during and following midlife [33].

An underlying motivation was expressed by participants who wished to “achieve longevity’” or “remain independent for as long as possible”. This finding concurs with a comparative study which conducted interviews with older people and found that the prospects of staying independent, maintaining current health status, improving physical balance, and retaining the ability to walk were key motivational factors for adherence to a group exercise program in the local community aiming to prevent falls [34].

While gaining improvements to physical fitness was the key motivational reason offered by participants for attending Aquarobics, all women confirmed they were also keen to access its mental and social benefits. Interestingly, recent research conducted with older adults concluded this group perceives exercise as “we time”, or as an opportunity to create and maintain social relationships. Whether it was spending time with a significant other, making new friends, or exercising to keep up with meaningful others, exercise served as a method of social interaction across both young and older age groups. Younger adults placed more weight on self-related motivations to exercise (“me time”), whereas older adults focused twice as much on exercise as a social experience (“we time”). While their research also involved focus groups, reference to ‘older’ participants included any adult over 59 years [35] (p. 714). Exercise participation has been found to reduce stress and anxiety, and improve mood [36,37].

Meeting social needs is a basic human requirement and feeing of connectedness to others and to community contributes to feelings of wellbeing and independence [38]. The greater sense of social connectedness made possible through the classes could be linked to the concept of caring, whereby members expressed care and concern for other participants when expressing feelings of caring about others and feeling cared about by others and gaining companionship or affection. Attending Aquarobics might help individuals experience greater feelings of belonging to a social group or community, and, therefore, less loneliness [2,39].

Several perspectives depict old age as a time of loneliness and rolelessness [40]; however, the findings of this case study support the health promoting aspects of participation leisure; that is, emphasizing what a person can do rather than what she or he cannot do, the importance of supportive environments, and the individual’s own perceived quality of her/his leisure experience [4,5]. In supporting the notion that social connectedness can be a positive and active part of older people’s lives, and reduce social isolation for this group of women, Aquarobics forms a leisure context creating an important space for women offering social connectedness, wellbeing, and empowerment [6,39]. Completing passive activities, such as watching TV, reading, or even gardening, while they might be considered enjoyable, are not associated with building social connectedness. Getting ‘out of the house’ and participating in leisure activities with others, such as Aquarobics, can form a health promoting support able to boost older people’s social connectedness and wellbeing [39,41].

It is very common in Australia for ‘indoor’ club venues, commercial gym and fitness center operators to offer lounge areas, cafes, and tables and chairs for members and participants to relax or socialize after ‘workouts’. However, it may not be as commonly accessible in rural or remote areas, or where the aquarobics class is conducted within a facility containing an Outdoor pool (only). It is, therefore, recommended to include future research testing experimentally the effects of aquarobics in a setting that features a social gathering space versus aquarobics in a setting that has no social gathering space. Any improvements to wellbeing could be measured for those participants meeting pre-class, during the class (all), and for that attributed to after-class socialization.

Further research is needed to better understand the potential health promoting benefits of participation in aquarobics by older women [9,10,11,12]. In particular, it will be helpful for each provider to explore what motivates their own particular group of participants to attend, as previous research has revealed these reasons may differ. Therefore, in order to provide the most ‘person-centered’ approach, it would help to consult with the group and try to tap into meeting this need through program delivery [42]. Finally, the pivotal role of friends, family, and doctors in the initiation of physical activity, plus the potential impact of the program’s instructor and other participants on the maintenance of these exercise behaviors are critical factors for investigation for us to gain insight into factors affecting motivation.

## 5. Conclusions

Achieving good physical health, mental health, and social connectedness are all linked to a healthy ageing process or satisfaction one is ‘ageing well’. The findings of this study suggest women are motivated to attend Aquarobics classes in order to gain improvements to subjective levels of fitness and wellbeing. According to the women, taking part helps them to feel more mobile and energized. It would be most helpful to better understand the role of the exercise leader and validate positive participant experiences with instructor expertise and experience, qualifications, attitudes, and beliefs. Due to the increased proportion of older people in the population, development of appropriate health promoting leisure programs readily available in the community is more important than ever.
